# Endoscopic Versus Open Carpal Tunnel Release: A Systematic Review of Outcomes and Complications

**DOI:** 10.7759/cureus.64991

**Published:** 2024-07-20

**Authors:** Ramkumar Rajapandian, Sajida Moti Wala, Esraa M Aledani, Essa A Samuel, Khoula Ahmad, Naelijwa J Manongi, Samia Rauf Butt

**Affiliations:** 1 Trauma and Orthopedics, California Institute of Behavioral Neurosciences and Psychology, Fairfield, USA; 2 Internal Medicine, California Institute of Behavioral Neurosciences and Psychology, Fairfield, USA; 3 Physical Medicine and Rehabilitation, California Institute of Behavioral Neurosciences and Psychology, Fairfield, USA; 4 Health Sciences and Public Health, California Institute of Behavioral Neurosciences and Psychology, Fairfield, USA; 5 Research, California Institute of Behavioral Neurosciences and Psychology, Fairfield, USA

**Keywords:** surgical procedure for carpal tunnel syndrome, carpal tunnel release, carpal tunnel syndrome, open carpal tunnel release, endoscopic carpal tunnel release

## Abstract

Carpal tunnel syndrome (CTS) is a condition that causes discomfort due to the compression of the median nerve in the wrist. Carpal tunnel release (CTR) is a surgical procedure that can help alleviate the symptoms of CTS. Two methods are commonly used for CTR: endoscopic carpal tunnel release (ECTR) and open carpal tunnel release (OCTR). The choice of method can impact surgical outcomes and potential complications. This review aims to compare the outcomes of both methods for individuals diagnosed with CTS. This systematic review analyzes the outcomes and potential complications of ECTR and OCTR for CTS. The study encompassed a comprehensive analysis of randomized controlled trials (RCTs) and meta-analyses comparing both methods. We searched for studies released between January 2012 and October 2023 on PubMed, Science Direct, and Google Scholar. The researchers assessed the quality of studies using the Cochrane risk of bias tool and the AMSTAR 2 (A Measurement Tool to Assess Systematic Reviews) tool. The study's scope included a range of outcomes and complications, such as symptom relief, functional recovery, grip strength, return to work, patient satisfaction, scar sensitivity, pillar pain, wound complications, nerve-related issues, morphological changes, and recurrence. The review analyzed 11 studies, including seven RCTs and four meta-analyses. These studies evaluated 4367 ECTR and 4107 cases of OCTR. The patients' ages ranged from 46 to 58, and the follow-up periods ranged from three to 24 months. The findings reveal that ECTR has comparable or better outcomes than OCTR, particularly in postoperative discomfort, functional recovery, grip strength, resumption of work, and patient satisfaction. Additionally, ECTR has lower levels of scar sensitivity, pillar pain, and wound-related complications than OCTR. However, ECTR carries a higher risk of reversible nerve injury. There were no substantial differences between the two techniques regarding other potential complications. Both ECTR and OCTR are safe and effective interventions for CTS. ECTR has benefits like faster recovery and improved cosmetic outcomes but requires higher technical proficiency and carries the risk of nerve injury. The choice of technique should consider patient preference, cost-effectiveness, and surgeon expertise.

## Introduction and background

The clinical presentation of carpal tunnel syndrome (CTS) involves experiencing discomfort, numbness, and reduced strength in the hand and arm, ascribed to the compression of the median nerve within the wrist region [1]. This crucial nerve runs from the forearm to the palm. The transverse carpal ligament can compromise its integrity. It is a fibrous band that forms the roof of the carpal tunnel [2]. A widespread ailment, CTS, impacts millions of individuals globally, with a prevalence of 3.8% and a lifetime risk of classic CTS symptoms estimated at 20% [3]. The prevalence of CTS within the general populace falls between 1% and 5%. CTS exhibits a higher occurrence among females than males, with a female-to-male ratio of 3:1 [4]. Surgery emerges as a viable recourse when non-surgical interventions, including splinting, medication, or injections, fall short of providing substantial relief [5].

Surgical intervention for CTS entails the surgical division of the transverse carpal ligament to alleviate pressure on the median nerve [6]. CTS is treated using two main surgical approaches: endoscopic carpal tunnel release (ECTR) and open carpal tunnel release (OCTR). ECTR represents a minimally invasive method using a small incision and a specialized endoscope equipped with a blade to sever the ligament within the carpal tunnel internally [7]. In contrast, OCTR adheres to the traditional approach, employing a larger incision and direct visual guidance to externally sever the ligament encompassing the carpal tunnel [8].

While both ECTR and OCTR have effectively mitigated CTS symptoms, a debate persists regarding which technique garners superiority regarding outcomes and complications. Some studies posit that ECTR offers advantages over OCTR, including swift recovery, reduced pain, improved cosmetic results, and heightened patient satisfaction [9]. Nevertheless, alternative studies contend that ECTR carries elevated risks of nerve injury, infection, and recurrence compared to OCTR, asserting that the disparities in outcomes lack clinical significance [10].

Hence, this systematic review seeks to juxtapose the outcomes and complications associated with ECTR and OCTR, leveraging the most robust evidence available in the literature. The review will encompass randomized controlled trials (RCTs) and meta-analyses appraising the two techniques in adult patients grappling with CTS. An array of outcome measures will be evaluated, including operation time, grip strength, sensory improvement, patient satisfaction, and complication rates. Additionally, the review will scrutinize the quality of the evidence and investigate potential sources of heterogeneity bias among the studies. By offering a comprehensive and critical appraisal of the comparative efficacy and safety of ECTR versus OCTR, the review is prepared to guide clinical decision-making and inform practice guidelines in treating CTS.

## Review

Methodology

This systematic review follows the guidelines and principles of the Preferred Reporting Items for Systematic Reviews and Meta-Analysis (PRISMA) 2020 [11].

Search Strategy

PubMed, PubMed Central (PMC), Medical Literature Analysis, Retrieval System Online (MEDLINE), Science Direct, and Google Scholar were used exclusively as research databases and search engines for this systematic review. The research utilizes CTS, open surgery, and endoscopy surgery. We used the Boolean term "OR" to combine the relevant concepts with specific keywords, as shown in Table 1

**Table 1:** PubMed search strategy with regular keywords

Concepts	Keywords	PubMed Search Builder
Endoscopy surgery	Endoscopy surgery, minimally invasive surgery	Endoscopy OR Minimal invasive procedure
Open surgery	Open surgery	Surgical Procedure, Operative
Carpal tunnel syndrome	Carpal tunnel syndrome, Median nerve neuropathy	Carpal tunnel syndrome OR Median Neuropathy

We utilized the same concepts as keywords to construct the Medical Subject Headings (MeSH) strategy, selecting subheadings such as adverse effects, complications, and surgery. Table 2 displays the results.

**Table 2:** MeSH strategy MeSH: Medical Subject Headings

Keywords	MeSH strategy
Endoscopy surgery	("Endoscopy/adverse effects"[Majr]) OR "Minimally Invasive Surgical Procedures/adverse effects"[Majr].
Open surgery	"Surgical Procedures, Operative/adverse effects"[Majr]
Carpal tunnel syndrome	(( "Carpal Tunnel Syndrome/complications"[Majr] OR "Carpal Tunnel Syndrome/surgery"[Majr] )) OR ( "Median Neuropathy/complications"[Majr] OR "Median Neuropathy/surgery"[Majr] )
Advanced Search Strategy	(((("Endoscopy/adverse effects"[Majr]) OR "Minimally Invasive Surgical Procedures/adverse effects"[Majr]) AND "Surgical Procedures, Operative/adverse effects"[Majr]) AND ( "Carpal Tunnel Syndrome/complications"[Majr] OR "Carpal Tunnel Syndrome/surgery"[Majr] )) OR ( "Median Neuropathy/complications"[Majr] OR "Median Neuropathy/surgery"[Majr]

Screening of Articles 

We gathered 1793 articles, removed duplicates, and evaluated the relevant papers based on their title, abstracts, and full-text content. Following this, we applied quality assessment tools to 20 research papers.

Inclusion Criteria 

The study centers on research involving adult patients aged 17 years and above undergoing ECTR or OCTR procedures. The study only includes studies published in English as full-text articles in peer-reviewed journals within the last 10 years, from 2012 to 2023. The study covers a variety of surgical methods, such as ECTR performed using the Agee, Chow, or Okutsu technique and OCTR performed using a standard or a mini-open incision. The study also incorporates a diverse selection of high-quality RCTs and meta-analyses that synthesize the results of multiple RCTs. The study excludes patients with secondary causes of CTS (e.g., diabetes, rheumatoid arthritis, and thyroid disorders) and those with prior wrist surgery.

Exclusion Criteria 

Excluded from this systematic review were studies that did not directly compare ECTR and OCTR. Instead, they focused on alternative interventions for CTS, such as conservative treatments or non-randomized studies. Also excluded were studies that lacked clear differentiation in reporting outcomes between ECTR and OCTR, including complications, functional recovery, and patient satisfaction. Other excluded studies had limited sample sizes, inadequate follow-up periods, insufficient surgical technique details, were non-peer-reviewed, or focused on pediatric patients or conditions affecting ECTR and OCTR outcomes. Finally, studies that did not have systematic literature search approaches, poorly described selection criteria, and excluded grey literature such as conference abstracts, dissertations, or reports.

Quality Assessment

In this systematic review, we included RCTs and meta-analyses using quality appraisal tools to evaluate bias risk during paper selection. We chose articles that met more than 70% of the criteria, and the accompanying tables illustrate their quality. Table 3 shows quality appraisal using the Cochrane bias assessment tool for randomized clinical trials. Table 4 shows quality appraisal using the AMSTAR (A Measurement Tool to Assess Systematic Reviews) checklist for meta-analysis.

**Table 3:** A quality appraisal using the Cochrane bias assessment tool + indicates yes, - indicates no, and ? indicates not clear.

Author	Type of study	Quality appraisal tool	Random sequence generation (selection bias)	Allocation concealment (selection bias)	Blinding of participants and personnel	Blinding of outcome assessment	Incomplete outcome data	Selection report	Other bias
Michelotti BM et al. (2018) [12]	Randomized clinical trial	Cochrane bias assessment tools	+	+	-	+	+	+	+
Ejiri S et al. (2012) [13]	Randomized clinical trial	Cochrane bias assessment tools	+	?	-	+	+	+	+
Kang H et al. (2012) [14]	Randomized clinical trial	Cochrane bias assessment tools	+	+	-	+	+	+	+
Oh W-T et al. (2017) [15]	Randomized clinical trial	Cochrane bias assessment tools	+	?	+	+	+	+	+
Chen Z et al. (2020) [16]	Randomized clinical trial	Cochrane bias assessment tools	+	?	-	?	+	+	+
Orak M et al. (2016) [17]	Randomized clinical trial	Cochrane bias assessment tools	+	+	+	+	+	+	?
Fernandes C et al. (2018) [18]	Randomized clinical trial	Cochrane bias assessment tools	+	+	+	+	+	+	?

**Table 4:** A quality appraisal using the AMSTAR checklist + indicates yes, - indicates no, and ? indicates not clear. AMSTAR: A Measurement Tool to Assess Systematic Reviews

Study characteristic	Kohanzadeh S et al. (2012) [19]	Vasiliadis HS et al. (2015) [20]	Li Y et al. (2020) [21]	Zuo D et al. (2015) [22]
Was an "a prior" design provided?	+	+	+	+
Was there duplicate study selection and data extraction?	+	?	+	+
Was the comprehensive literature search performed?	+	+	+	+
Was the publication status (i.e., grey literature) used as an inclusion criterion?	+	+	+	_
Was the list of studies (included and excluded) provided?	+	+	+	+
Are the characteristics of the included studies provided?	+	+	+	+
Did they evaluate and document the scientific quality of the included studies?	+	+	+	+
Was the scientific quality of the included studies appropriately considered when formulating conclusions?	+	+	+	+
Were the methods used to combine the findings of studies appropriate?	?	+	+	+
Was publication bias likelihood assessed?	?	+	+	+
Was the conflict of interest stated?	?	+	+	+

Result

**Figure 1:**
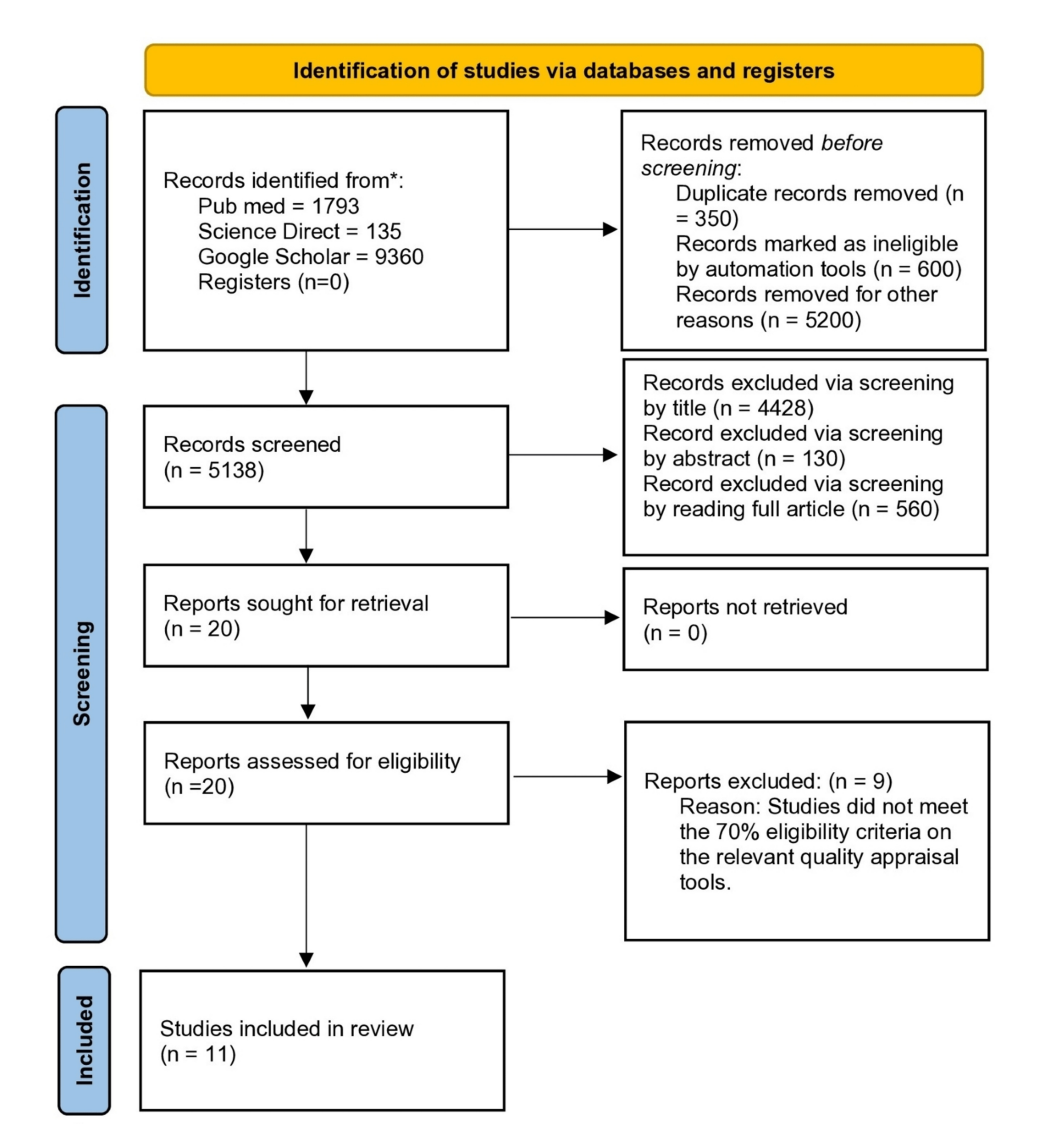
PRISMA 2020 flowchart depicting the process for article selection ^* ^studies included based on predefined inclusion criteria established before screening PRISMA: Preferred Reporting Items for Systematic Review and Meta-Analysis

We searched four databases electronically to look for relevant studies. Initially, we found 11288 articles related to our topic. Afterward, automation tools removed 350 duplicates and 5800 papers because of ineligibility. This number was further reduced to 20 after the screening, based on inclusion/exclusion criteria and relevant title, abstract, and full-text reading. Finally, the quality assessment tools assessed the bias in the studies [23,24]. Ultimately, we finalized 11 articles and removed the remaining nine due to poor quality. Figure 1 exhibits the search strategy used to conduct this review in a PRISMA flowchart [11].

Table 5 summarizes all randomized controlled studies, and Table 6 summarizes the characteristics of all meta-analysis studies.

**Table 5:** Summary of RCT OCTR: open carpal tunnel release; ECTR: endoscopic carpal tunnel release; BCTQ: Boston carpal tunnel syndrome questionnaire; DASH: disabilities of the arm, shoulder, and hand; MECTR: modified carpal tunnel syndrome; VAS: visual analog pain scale; RCT: randomized controlled trials

Author and year of publication	Country	Sample size	Incision size	Technique	Follow-up and complication	Conclusion
Michelotti BM et al. (2018) [12]	USA	ECTR: 13 hands OCTR: 17 hands	ECTR: 1.5-2 cm; OCTR: 3 cm	The surgeon uses a single portal for ECTR and incises for OCTR along the radial border of the ring finger over the transverse carpal ligament	One week, six weeks, three months, and six months postoperatively. There is no compilation	Patients preferred and were highly satisfied with ECTR and experienced faster grip and pinch strength recovery than OCTR
Ejiri S et al. (2012) [13]	Japan	ECTR: 51 hands OCTR: 50 hands	ECTR: not reported; OCTR: 3 cm	ECTR: Okutsu technique. OCTR: palmar incision method	One week, two weeks, four weeks, eight weeks, and 12 weeks. ECTR might cause temporary nerve dysfunction, usually resolving within six months post-op	The two techniques showed similar outcomes and complication rates during follow-up. However, the endoscopic method had shorter operation times and less scar tenderness than the open approach
Kang H et al. (2012) [14]	Korea	52 patients on bilateral CTS, ECTR: 26 hands, OCTR: 26 hands	ECTR: 1.5 cm; OCTR: 1.5 cm	ECTR: Agee’s technique. OCTR: the mini-open approach	ECTR showed transient worsening neuropathy symptoms one, two, four, eight, and 12 weeks after surgery	The BCTQ and DASH questionnaires revealed comparable outcomes between the two procedures. Nevertheless, a majority of patients expressed a preference for ECTR over the mini-open procedure
Oh W-T et al. (2017) [15]	South Korea	ECTR: 35 patients, mini OCTR: 32 patients	ECTR: 1.5 cm for each port site; mini OCTR: 1.5 cm proximal and 3 cm distal to the wrist creases	ECTR: Agee’s technique. OCTR: mini-open approach	Neither group experienced significant surgical complications preoperatively or 24 weeks postoperatively	At the 24-week postoperative assessment, no substantial variations were observed in subjective outcomes or ultrasound evaluations assessing morphological changes between the groups
Chen Z et al. (2020) [16]	China	Modified ECTR: 48 patients, OCTR: 46 patients	Modified ECTR: 1 cm; OCTR: 6 cm	MECTR: Single portal technique. OCTR: traditional open surgery	No complications were noted in one week, one month, three months, and six months after surgery	No significant difference. Interestingly, METR exhibited no complications related to nerves
Orak M et al. (2016) [17]	Turkey	ECTR: 21 patients OCTR: 28 patients	ECTR: 1 to 1.5 cm; OCTR: 3 to 4 cm	ECTR: Chow’s technique. OCTR procedure entails an incision along the thenar crease, positioned distally to the Kaplan oblique line	One week, two weeks, four weeks, and 24 weeks postoperatively. In the study, one ECTR patient had a flexor digitorum superficialis injury, while a single OCTR patient reported ongoing scar-related pain six weeks after surgery	Preoperative and postoperative questionnaires showed similar outcomes for both procedures, yet ECTR patients reported less postoperative pain than those with OCTR
Fernandes C et al. (2018) [18]	Brazil	ECTR: 15 hands OCTR: 15 hands	ECTR: 2 to 3 cm; OCTR: 4 cm +/- 1 cm extension	ECTR involves a single portal technique, while OCTR requires a longitudinal incision over the external border of the hypothenar eminence	No complications were recorded two weeks, one month, three months, or six months after surgery	Open and endoscopic surgeries showed similar postoperative results on the Boston Questionnaire, VAS, and grip strength. Additionally, OCTR patients displayed improved three-digit grip strength six months after surgery

**Table 6:** Summary of the Meta-analysis OCTR: open carpal tunnel release; ECTR: endoscopic carpal tunnel release; CTS: carpal tunnel syndrome

Author and year of publication	Study design	Number of studies	Number of patients included	Complication	Conclusion
Kohanzadeh S et al. (2012) [19]	Meta-analysis	22 studies	ECTR: 1,189 patients; OCTR: 1,187 patients	The ECTR group experienced more temporary nerve damage (2.2%) than the OCTR group (1.2%). However, the two groups did not differ significantly in lasting nerve injury	The study found that ECTR performed better than OCTR in eight measures, except for complications. The study recommended ECTR over OCTR, especially for skilled surgeons
Vasiliadis HS et al. (2015) [20]	Meta-analysis	28 studies	ECTR: 1317 patients; OCTR: 1315 patients	Pillar pain, scar-related issues, and postoperative infections were reduced by 76% in cases of ECTR	The research demonstrated comparable safety between ECTR and OCTR for CTS treatment, with no notable disparities in severe complications, reoperations, or symptom recurrence. ECTR also involved shorter recovery periods than open surgery. They strongly recommend surgeons consider switching to OCTR if difficulties arise during ECTR to mitigate potential complications
Li Y et al. (2020) [21]	Meta-analysis	28 studies	ECTR: 964 patients; OCTR: 764 patients	Temporary nerve injury incomplete release is more frequent in ECTR, and open surgery has more issues with infection, scar tenderness, and pillar pain	ECTR improved satisfaction and critical pinch strength and reduced scar-related issues compared to OCTR in CTS patients. However, it increased temporary nerve injury without affecting permanent nerve injury. The study proposed ECTR as a potential treatment for rapid recovery of daily functions in CTS patients
Zuo D et al. (2015) [22]	Meta-analysis	13 studies	ECTR: 688 patients; OCTR: 627 patients	ECTR has a higher incidence of reversible nerve injury, while OCTR is associated with scar tenderness, pillar pain, wound infection, hematoma, and reflex sympathetic dystrophy	No discernible statistical disparity was observed between the two groups of patients in terms of overall complication rate, subjective satisfaction, the time required for resumption of occupational duties, postoperative grip, pinch strength, and operative duration. However, postoperative pain is significantly lower in ECTR

Discussion

CTS is a prevalent condition that substantially affects patients’ quality of life. Primary surgical interventions encompass OCTR and ECTR. The selection between the endoscopic and open approaches remains controversial [25-27]. This systematic review aims to comprehensively compare the outcomes and complications associated with these techniques, aiding surgeons in transparently discussing potential complications with patients. This comparison assists in selecting the optimal management aligned with individual patient preferences. The review emphasizes essential metrics encompassing reversible nerve injury, postoperative pain, operative duration, grip strength, Boston Carpal Tunnel Questionnaire (BCTQ) scores, digital sensation, patient contentment, return-to-work duration, and diverse complications.

In addition to covering all aspects, this review discusses the techniques employed in various studies. Some studies used the one-portal technique (Agee’s), while others employed the two-portal techniques like Chow’s and Okutsu [28-30]. Notably, no RCTs have been conducted directly to compare these techniques; however, no apparent differences were observed in the outcomes. Nonetheless, it is worth noting that the two-portal technique showed a higher incidence of scar-related complications and improved surgeons’ visibility during the procedure [12].

Reversible Nerve Injury

One of the primary complications associated with carpal tunnel release (CTR) surgery is reversible nerve injury, leading to transient loss of sensation or movement in the fingers or hand. This injury can be attributed to direct trauma, compression, or elongation of the median nerve or its extensions during the surgical procedure. Studies suggest that ECTR poses a comparatively higher risk of reversible nerve injury than OCTR. Most investigations did not consistently observe statistical significance. In a meta-analysis by Zuo D et al., the aggregated risk ratio for reversible nerve injury between ECTR and OCTR was 2.38 (95% CI: 0.98, 5.77) [22].

In comparison, another meta-analysis by Li Y et al. reported a ratio of 1.67 (95% CI: 1.37 to 17.25) [21]. Several studies suggest a somewhat increased risk associated with ECTR compared to OCTR. It is important to note, however, that nerve injuries observed in these cases typically showed recovery within three to six months [12-14,17-22]. Notably, Chen Z et al. reported modified ECTR for CTR with no observed nerve injuries, indicating a potential technique modification [18]. However, more trials are crucial to evaluate the efficacy of this modified approach. The heightened risk of nerve injury in ECTR could stem from factors like limited nerve visibility, instrument sharpness, proximity, and surgeon expertise.

Postoperative Pain

This is a crucial factor after CTR surgery. A review found that ECTR causes less postoperative pain than OCTR, especially in the early postoperative period. ECTR had significantly less postoperative pain than OCTR at most time points, except for six or 12 months after the surgery. The reason for less postoperative pain in ECTR is the smaller incision, less tissue damage, less scar formation, and less inflammation [12,13,18,22].

Common Complications

Post-surgery CTR poses various complications like scar tenderness, pillar pain, infection, hematoma, wound issues, and complex regional pain syndrome (CRPS). While ECTR and OCTR releases share similar overall complication rates, their specific complications vary. Chen et al. (2020) reported a 5.8% overall complication rate [16]. Meta-analyses and RCTs consistently show no statistically significant difference in the combined risk of complications between ECTR and OCTR [12,14-22]. ECTR commonly leads to reversible nerve injury and scar tenderness, while OCTR tends to cause scar tenderness and pillar pain. Incidences of infection, hematoma, wound issues, and CRPS were rare in both groups. Differences in surgical approaches, healing processes, and patient traits likely explain these complication disparities.

Patient Satisfaction and Preference

The primary objective of CTR procedures is to ensure patient contentment and inclination. In a collective examination of three studies by Li et al., Zuo et al., and Michelotti BM et al., notable distinctions emerged in patient satisfaction and preference favoring ECTR over OCTR [12,21,22]. Patient satisfaction exhibited consistently high levels in both groups across various assessment periods, except in cases where scar-related complications led patients to choose ECTR [14-20]. The convergence of patient satisfaction and preference possibly arises from both methodologies’ comparable effectiveness and safety profiles, the elevated patient expectations and acceptance levels, and the significant influence wielded by the surgeon’s recommendations.

Operative Time, Grip Strength, BCTQ Scores, and Digital Sensation

The study found no significant disparities between ECTR and OCTR in surgical duration, grip strength, BCTQ scores, and digital sensation. Both methods were equally effective in alleviating symptoms and enhancing functionality in CTS patients. Insights from studies by Michelotti BM et al. and Ejiri et al. highlighted favorable outcomes in grip strength and muscle recovery for the ECTR group, possibly due to reduced tissue damage during the procedure compared to OCTR [12,13,31,32]. However, in Fernandes et al., the OCTR group showed improved three-digit grip strength after six months, with no notable differences in longer-term assessments [18]. Therefore, other factors beyond functional outcomes may influence ECTR and OCTR decisions.

Cost Considerations

It is important to note that ECTR is generally more expensive than OCTR due to the higher cost of endoscopic equipment and disposable instruments [32,9]. The cost-effectiveness of ECTR may vary depending on several factors, such as the availability of resources, the number of cases, and whether patients are willing to cover the costs associated with the procedure.

Miscellaneous

Numerous studies have offered illuminating perspectives comparing ECTR and OCTR. Ejiri et al. (2012) suggested a potential rise in carpal tunnel pressure with ECTR, especially in severe CTS cases, which might exacerbate postoperative symptoms and lead to transient, reversible nerve injuries, and contrasting OCTR outcomes. Hence, they advocate for OCTR in patients exhibiting abductor pollicis brevis-distal latency >/10 ms [13]. Chen et al. demonstrated a complete absence of median nerve injuries using the METCR technique, employing specialized axillary endoscopic equipment without a metal mantle tube insertion during the procedure. Additionally, they applied the fat suspension technique to expand the operative space [16]. However, further RCTs are necessary to assess the efficacy of the modified ECTR technique precisely. Kang et al. employed the mini-open incision and observed no difference but reduced incidence of common complications with the open technique [14]. Furthermore, Kohanzadeh et al. (2012) advocate for experienced surgeons to perform ECTR to minimize nerve-related complications, underscoring the pivotal role of surgical expertise in ensuring favorable surgical outcomes [19].

Limitations

The diversity among the included studies introduces limitations to this systematic review. Variability exists in study designs, sample sizes, follow-up durations, surgical techniques, outcome assessments, and study quality. Interpretation of the results requires caution, and additional high-quality RCTs warrant further validation.

## Conclusions

In this systematic review, comparable outcomes and complications were observed between ECTR and OCTR in the management of CTS. Nevertheless, each technique presents its own set of advantages and disadvantages. ECTR demonstrates higher satisfaction rates, more incredible key pinch strengths, quicker return to work durations, and fewer scar-related complications. However, it is also correlated with elevated rates of transient nerve injuries and increased costs compared to OCTR. Conversely, OCTR shows lower rates of transient nerve injuries and reduced costs, albeit with lower satisfaction rates and a higher occurrence of scar-related complications than ECTR. Both methods prove equally effective in alleviating CTS symptoms and enhancing patient functionality. Consequently, the surgical approach should consider the patient's informed decision, which includes a comprehensive understanding of potential outcomes and complications, the surgeon's expertise, resource availability, and the case's complexity.
